# Needed in Psychology: Theoretical Precision

**DOI:** 10.5964/ejop.v14i1.1602

**Published:** 2018-03-12

**Authors:** Jaan Valsiner

**Affiliations:** a Department of Communication and Psychology, Aalborg University, Aalborg, Denmark

Psychology is a strange science. Its practitioners adhere to the deep belief that claiming it to be “empirical science” makes it into a solid science. The glorious image here is that of the active and diligent data collector who follows all the rules of making scientific inference from some ever larger data sets. The inductive pathway to knowledge becomes glorified. The belief in quantification of phenomena into data and the belief that the data accumulation that follows (“big data”) leads to solid basic knowledge becomes an accepted belief. “Evidence based science” is an inductive exercise based on collections, rather than careful theory-based entrances into crucially relevant human psychological worlds. While glorifying “the evidence” we forget to ask: what kind of evidence is needed for what kind of theoretically relevant question? It is as if the data—accumulated to huge data bases—will start to “speak for themselves” and psychology’s future as a “true” science will be ensured.

I happen to doubt this scenario of a positive future for psychology as a data-accumulative science. There are good reasons for my doubts. Psychology was—like chemistry—in a state in late 18^th^ century that made Immanuel Kant deny the futures of both as sciences. Neither were seen by him as open to mathematical clarity of pure thought. He was wrong about chemistry that from the 1830s onwards developed its elaborated system of multitude of formal languages (Valsiner, 2012, Chapter 19) and generalized framework of organization in the form of Mendeleev’s Periodicity Table. Yet Kant was right about psychology, that has substituted efforts to build linkages with basic mathematics (Rudolph, 2013) in favour of applied mathematics in the form of statistics, and allowed the latter to take over the role of theoretical argumentations. Tools—statistical techniques like analysis of variance—have been turned into theories (Gigerenzer, 1991). The result is stagnation in the methodological advancement of the field (Toomela & Valsiner, 2010), stagnation that reaches even to the contemporary favourite area of neuroscience (Toomela, in press). Psychology resembles a powerful machine that is set to rush ahead in high gear—but cannot remove its self-set brakes.

I belong to the rare species of theoretical psychologists for whom the often used label “contemporary research” means not the work of the past two, but rather that of the past 200 years. From that somewhat extended perspective, it becomes evident that one of the limitations of our contemporary psychology is the erosion of the term “empirical”. Back in the times when our contemporary psychology parted with its philosophical predecessors—the 1870s—that term meant experiential (Brentano, 1874). Experience—rather than accumulated data—were the source of empirical investigation. In the century that followed, it is this focus on experience as a crucial base for phenomena that got lost (Cairns, 1986; Molenaar, 2004).

I see a way out in the restoration of this original notion of “empirical” as direct, unique, and complex experience, as the focus of theoretically based empirical research. Some examples already exist (Lehmann & Valsiner, 2017). This has implications to the kind of data that psychology needs to advance its *theoretical* precision—a quality that is needed for making sense of phenomena of experience. We are used to think of the precision of the instruments we utilize—together with the kind of data these instruments can provide. I claim the same demand for precision is applicable in the theoretical realm.

Psychology has very limited theoretical precision. Consider an example:

“Variable X *may be linked* with Variable Y”

This claim has low precision—the kind of *link* implied is not elaborated (there are many possibilities of how a link can be conceptualized) and the “may be” is immediate contrasted with “need not be”. A modified version of a similar statement does not fare better:

“Variable X *is found to be significantly linked* with Variable Y”

Here the link is specified by the vague mentioning of significance. That term is ambiguous—it confounds statistical significance (which is well defined) with loosely referenced *general* significance. That kind of significance needs to be further specified. For instance, it can take the form of a “fixed link” (e.g., two electronic gadgets linked with a wire), or of a dynamically stable process of mutuality (X feeds into Y and Y reciprocally feeds into X). It is not clear if the presumed link is independent of other links, or catalyzed by some external conditions (see Cabell & Valsiner, 2014, on catalytic models). In sum, it is precisely where the inductive generalization ends (something is significantly linked with something else) where the theoretical precision needs to begin. One rarely sees that in recent psychology.

I would like to put forth a call for establishing *nanopsychology*—psychology where the empirical data base is purified to include humanly important existential experiences (focus on selected data that are given in their minimalistic form) which are then put to rigorous theoretical scrutiny. For such innovation, we start from carefully pre-selected and inevitably unique psychological phenomenon. It can be a relevant rupture in a person’s life course (Zittoun, 2006). Or it can start from a single poetic instant (Lehmann, 2015) in experiencing the serene moment of silence in a Norwegian countryside. Likewise, the phenomena of personal importance can be the moments of orgasm—deeply subjective and rarely described (Matte Blanco, 1998, pp. 441-442). These are all minimal moments of deep experiences that lead our general life courses into the silence of our subjectivity. Nanopsychology starts from these—very small, unique, and transient—particular phenomena.

But, of course, our deep subjective life-worlds are socially guided. Evidence for such guidance can be massive—our environments are saturated by redundant messages in different forms suggesting how to feel, think, and act. Yet the integration of these socially suggestive representations into my personal life-world is unique to me, and happens at a moment of affective synthesis (Vygotsky, 1971—on Ivan Bunin’s *Legkoe Dykhanie*). Where and when precisely such synthesis would happen remains unpredictable—but the massive social guidance of any person towards it is socially visible. If we dare to look, of course.

**Figure 1:**
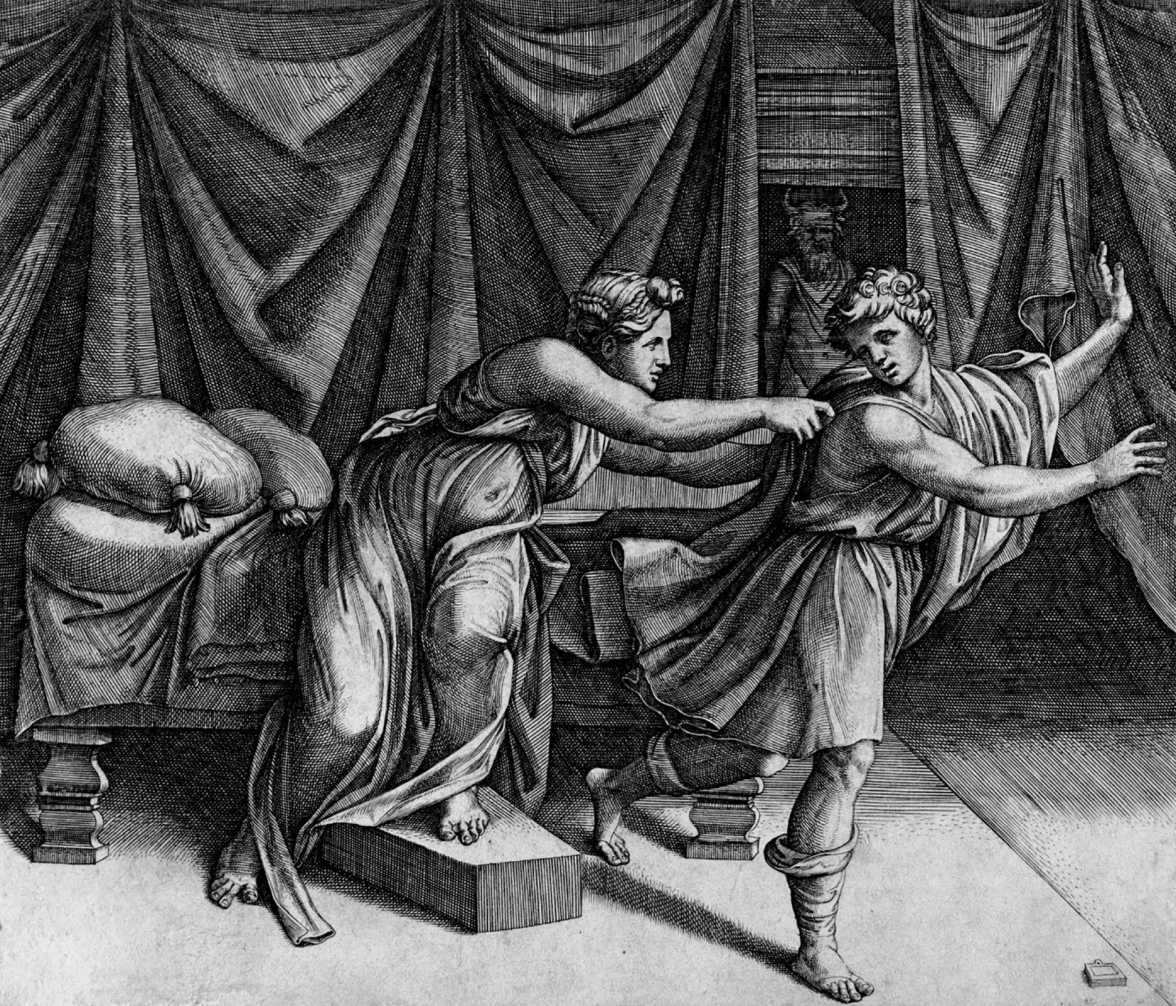
Marcantonio Raimondi’s engraving JOSEPH AND POTIPHAR WIFE (1520) (work in the public domain).

Let me illustrate this general idea—massive social guidance for deeply personal feelings that are expected to integrate into basic personal values at some unique moment—as an example of unique data point that needs thorough theoretical analysis. The Persian tale—which also found its reflection in the Bible (Book of Genesis) of the desires of Zulaikha and resistance by Joseph, her husband Potiphar’s maidservant—is a story of love and revenge, as well as of adultery and honorable respect. The story may be partially a myth of 6^th^ century BC possible real event in Egypt that moved from oral folklore to written texts of Islamic and Christian kinds over the first millennium of our age, and became a widely popular topic for artists’ depictions in Europe in the 16^th^ to 18^th^ centuries AD. An early example is given in Figure 1.

Joseph is Potiphar’s household slave who is given the role of the head of his household and, within the household, Potiphar’s wife Zulaikha becomes drawn to Joseph and wants him to be her lover. Joseph—dedicated to Potiphar—refuses. Zulaikha, in her anger, accuses Joseph for making advances on her, and Potiphar arrests Joseph on the basis of this accusation. The story continues with Joseph being pardoned later, but it is the single specific moment from the story—where Zulaikha reaches out towards Joseph in her desire for him (and Joseph resists or runs away) that was the recurrent theme to depict by artists.

This moment depicted in the scene (Figure 1) encodes the two universal notions of DESIRE and RESISTANCE into the process of interpreting one pictorial image. By picturing the tension in the unity of the two in the picture it leads to further consideration of sign complexes of LOVE, ADULTERY, HONOR – all constantly in use in human lives. An elaborate and precise theoretical analysis of the *multiple pathways* the affective guidance it would trigger under different interpretations would give us a glimpse into the field of possibilities that can be activated by a recipient of the social suggestion.

Without any interpretive guidance the picture is simple—a woman reaches out towards a man who is in the process of escaping from her. Different pathways emerge on the basis of co-interpretation of the picture. The first pathway is the moralistic tension of ADULTERY <> HONOR. Joseph’s resistance to Zulaikha’s advance creates a border that publicly suppresses DESIRE through HONORABLE RESISTANCE. Joseph cannot succumb to Zulaikha’s advances given his role in Potiphar’s household and given the adulterous nature of her DESIRE. Note that this contextual condition is not encoded into the picture; it needs to be complemented by the recipient’s knowledge of the story. HONOR leads to RESISTANCE which blocks the DESIRE (of hers) since that DESIRE was to cross the border of marital relations (ADULTERY). This structure of social representations used to build the interpretation sets Joseph up as a hero and Zulaikha as a villain (who, in the continuation of the story—not in the painting—accuses Joseph for making advances *on her*—breaking the HONOR of marriage). A second pathway is completely different. Zulaikha is considered deeply in LOVE (Shafak, 2005) with Joseph and her DESIRE is an act of heroic defiance of the bounds of marriage. ADULTERY here is an act of courage, and Joseph’s RESISTANCE—an act of cowardice hidden behind his social commitment to Potiphar.

Neither of these two pathways are in the picture itself, but both are possible in the unique personal acts of co-interpreting the picture based on the tacit knowledge about the story. There may be still other pathways not considered here. The co-interpretive set is much richer than its immediate input—the picture. Our theoretical analysis charts out all of that set, specifying the particular ways in which the social representations form a new Gestalt of meanings. Each version of that new Gestalt leads to different ways of “feeing into” the picture. The picture is a catalyst to keep the tensions of the story reverberating in the human minds.

Nanopsychology starts from the particular relevant event in human lives—tension at crossing some border—and ends up finding general principles in the selected “minimal data”. This is possible only on the theoretical basis that suggests what kinds of data are relevant and how to find them. This, in turn, requires theoretical precision well ahead of any empirical investigation.

**Jaan Valsiner** *Aalborg, February 2018*
